# Salicin alleviates periodontitis via Tas2r143/gustducin signaling in fibroblasts

**DOI:** 10.3389/fimmu.2024.1374900

**Published:** 2024-03-28

**Authors:** Zhiying Zhang, Zhiyan Zhou, Jiaxin Liu, Liwei Zheng, Xian Peng, Lei Zhao, Xin Zheng, Xin Xu

**Affiliations:** ^1^ State Key Laboratory of Oral Diseases & National Center for Stomatology & National Clinical Research Center for Oral Diseases & Department of Cariology and Endodontics, West China Hospital of Stomatology, Sichuan University, Sichuan, Chengdu, China; ^2^ Department of Cariology and Endodontics, School and Hospital of Stomatology, Cheeloo College of Medicine, Shandong University & Shandong Key Laboratory of Oral Tissue Regeneration & Shandong Engineering Research Center of Dental Materials and Oral Tissue Regeneration & Shandong Provincial Clinical Research Center for Oral Diseases, Shandong, Jinan, China; ^3^ State Key Laboratory of Oral Diseases & National Center for Stomatology & National Clinical Research Center for Oral Diseases & Department of Pediatric Dentistry, West China Hospital of Stomatology, Sichuan University, Sichuan, Chengdu, China; ^4^ State Key Laboratory of Oral Diseases & National Center for Stomatology & National Clinical Research Center for Oral Diseases & Department of Periodontology, West China Hospital of Stomatology, Sichuan University, Sichuan, Chengdu, China

**Keywords:** taste/taste physiology, periodontal disease(s), fibroblast(s), chemokines, bone loss, infectious disease(s)

## Abstract

**Introduction:**

Cells expressing taste signaling elements in non-gustatory tissues have been described as solitary chemosensory cells (SCCs) or tuft cells. These “taste-like” cells play a critical role in the maintenance of tissue homeostasis. Although the expression of SCC markers and taste signaling constituents has been identified in mouse gingivae, their role in periodontal homeostasis is still unclear.

**Methods:**

Public RNA sequencing datasets were re-analyzed and further validated with RT-PCR/qRT-PCR and immunofluorescent staining to explore the expression of TAS2Rs and downstream signaling constituents in mouse gingival fibroblasts (MGFs). The specific action of salicin on MGFs via Tas2r143 was validated with RNA silence, heterologous expression of taste receptor/Gα-gustducin and calcium imaging. The anti-inflammatory effects of salicin against LPS-induced MGFs were investigated in cell cultures, and were further validated with a ligature-induced periodontitis mouse model using Ga-gustducin-null (Gnat3^−/−^) mice.

**Results:**

The expression of Tas2r143, Gnat3, Plcb2, and TrpM5 was detected in MGFs. Moreover, salicin could activate Tas2r143, elicited taste signaling and thus inhibited LPS-induced chemokines expression (CXCL1, CXCL2, and CXCL5) in MGFs. Consistently, salicin-treatment inhibited periodontal bone loss, inflammatory/chemotactic factors expression, and neutrophil infiltration in periodontitis mice, while these effects were abolished in Gnat3^−/−^ mice.

**Discussion:**

Gingival fibroblasts play a critical role in the maintenance of periodontal homeostasis via “SCC-like” activity. Salicin can activate Tas2r143-mediated bitter taste signaling and thus alleviate periodontitis in mouse, indicating a promising approach to the resolution of periodontal inflammation via stimulating the “SCC-like” function of gingival fibroblasts.

## Introduction

Periodontitis is a disease caused by chronic infection and destruction of periodontal tissue. It is the accumulation of a dysbiotic biofilm and the subsequent immune reaction that triggers periodontal inflammation and the subsequent loss of attachment and alveolar bone resorption (1). In severe cases, it causes tooth loosening and detachment, making it one of the major diseases that endanger human oral health. In addition, periodontitis is closely related to systemic diseases such as diabetes, cardiovascular and cerebrovascular diseases, rheumatoid arthritis, adding increasing socioeconomic burden to the society (2).

Gingival fibroblasts are the major cellular component of gingival tissue. Gingival fibroblasts not only synthesize extracellular matrix to promote wound healing, but are also involved in the antimicrobial response of periodontium (3). In the development of periodontitis, gingival fibroblasts secret inflammatory cytokines that are involved in the regulation of leukocyte proliferation, granulocyte migration and complement activation (4–6). In the later stage of inflammation, sustained release of cytokines and excessive immune cell recruitment can cause unresolved inflammation, contributing to periodontal destruction. Previous studies have shown that, chemokines are mainly secreted by immune cells such as neutrophils, and inhibition of chemokines can alleviate periodontal destruction (7, 8). However, recent scRNA-seq study has shown that fibroblasts are particularly transcriptionally active in the production of chemokines with potential to recruit neutrophils (CXCL1, 2, 5, 8) and other leukocytes (e.g. CXCL12, CXCL13, CCL19) (3). This suggests the important role of gingival fibroblasts in the development and progression of periodontitis.

Recent studies have identified a special population of cells in non-gustatory tissues that express bitter taste receptors (TAS2Rs) and taste transduction elements such as Gα-gustducin (GNAT3), PLCβ2, TRPM5, etc (9, 10). These taste-like cells, usually described as solitary chemosensory cells (SCCs) or tuft cells, reside in a variety of tissues including respiratory tract, gastrointestinal mucosa, urethra, heart, and gingiva, etc (11). Recent studies have demonstrated that SCCs can sense and respond microbial metabolites to regulate host-microbe interactions (12). Our previous studies have demonstrated the expression of taste transduction elements in mouse periodontal tissue, and defect in the bitter taste transduction (Gnat3^–/–^) aggravated periodontal inflammation and alveolar bone destruction in mice (13). In addition, we have recently demonstrated that bitter compound salicin can activate TAS2R16 and decrease the cAMP level in human gingival fibroblasts, thus suppressing NF-κB signaling cascade and antagonizing LPS-induced cytokine expression in vitro (14). However, whether this bitter signaling-dependent inflammation resolution takes effect in vivo still needs validation. Therefore, the current study aimed to validate the expression of Tas2rs and taste transduction elements in mouse gingival fibroblasts (MGFs), and investigate their role in regulating periodontitis under salicin stimulation in vivo. The results are expected to demonstrate the regulatory role of gingival fibroblasts in periodontitis via SCC-like activity, and advance our knowledge in the development and resolution of periodontal inflammation.

## Materials and methods

### Re-analysis of public datasets

We retrieved and re-analyzed sequencing data from Gene expression omnibus (GEO) database (https://www.ncbi.nlm.nih.gov/gds). We screened the RNA-Seq/SC-RNA- Seq data of healthy/untreated gingival and MGF samples, and included RNA-Seq datasets GSE118300, GSE118166, GSE118952, GSE178616, GSE186882, GSE228021, and scRNA-seq datasets GSM5672499, GSM5600724, GSM7767515 for re-analysis. We counted the expression of bitter taste receptors and taste transduction elements in each dataset based on RNA Seq data. According to the study by Williams et al. (3), we performed homologous transformation of the target gene from human to mouse and annotated MGFs into 6 cell subtypes in the scRNA-seq datasets. The average expression level of specific gene set for each cell subtype in MGFs was obtained using the AverageExpression function. Heatmap of the transposed data matrix was drawn with cell subtypes as rows and genes as columns. In addition, the percentage of mitochondrial and red blood cell genes was calculated for each cell as a quality control indicator. The NormalizeData function was used to identify and standardize variant feature genes, and PCA was run for preliminary dimensionality reduction analysis. DimPlot was used to plot PCA results to visualize the variability of the data. Harmony algorithm was introduced to handle batch effects, another dimensionality reduction was performed, and ElbowPlot was used to select the optimal dimension. DimPlot was used to draw UMAP graphs, the data were encoded with color according to organizational and cell subtypes to demonstrate the high-dimensional structure and clustering effectiveness.

### Cell culture

To extract primary mouse gingival fibroblasts (MGFs), six 7-8-week-old SPF C57BL/6 male mice were selected. All experimental procedures involving animals were conducted in accordance with the guidelines approved by the Ethics Committee of West China Hospital, Sichuan University (WCHSIRB-D-2021-071). Bilateral maxillary buccal gingival tissue of the mice was taken under a stereomicroscope (Olympus, SZX10) and soaked and washed in Hank’s Balanced Salt Solution (HBSS, Hycolne) at 4°C containing 10% penicillin streptomycin solution (PS, Hycolne) for 20 minutes. The gingival connective tissue was cut into small blocks using ophthalmic scissors. Dulbecco’s modified eagle medium (DMEM, Gibco) containing 1% penicillin streptomycin solution (Hyclone) and 10% fetal bovine serum (FBS; Gibco) was used to gently blow and mix. The tissue blocks were transferred to T25 culture bottles (Corning) and cultured at 37°C and 5% CO_2_. The culture medium is changed every 3 days and the 3rd to 5th generation cells were used for the next experiment.

Human embryonic kidney 293 (HEK293) PEAKrapid cells were obtained from ATCC and cultured in Opti-MEM (Thermo Fisher Scientific Inc.) with 4% fetal bovine serum at 37°C and 5% CO_2_.

### A mouse model of periodontitis

The design and production of Gα-gustducin-null (Gnat3^−/−^) mice (kindly provided by Peihua Jiang, Monell Chemical Senses Center) are as described earlier (15), and the genetic background of mice is C57BL/6J. All mice were placed in the same environment, subjected to a 12 hour light dark cycle under specific pathogen-free conditions, and freely obtained food and water. 8-week-old male mice with comparable weight (25±5 grams) were randomly divided into 4 groups (i.e. wild type (WT) or Gnat3^−/−^ mice treated with salicin or vehicle, n=12 per group). The study design adhered to the ARRIVE guidelines for preclinical studies. A simplified ligature-induced periodontitis (LIP) mouse model was established according to the method reported previously by Julie Marchesan et al (16). A 5-0 surgical suture was ligated between the first (M1) and second molars (M2) in the left maxillae of mice after anesthetized by intraperitoneal injection of 2% pentobarbital sodium solution (50 mg/kg). The right side without ligation was used as negative control to determine whether the model of periodontitis has been successfully constructed. A small cotton swab was dipped into 100 mg/kg salicin or an equivalent volume of saline (vehicle), and was then applied repeatedly to the left upper cheek and palatal gingiva of the experimental mice twice a day. The mice were refrained from water for 1 hour after application. After 7 days of applying the medication, the experimental mice were euthanized, and the buccal gingival tissue of left maxilla M1-M2 as well as bilateral maxillary bones and teeth were collected under a stereomicroscope (Olympus, SZX10).

### Immunofluorescent staining

The cultured MGFs were fixed with 4% paraformaldehyde for 10 minutes, and then permeated with 0.5% Triton X-100 at room temperature for 10 minutes. After blocking with 1% BSA for 1 hour, the cells were incubated overnight with the specific primary antibody at 4°C for vimentin (1:100; Wanleibio, WL01960), Gnat3 (1:100; Invitrogen, PA5-50670), TrpM5 (1 µg/mL; Invitrogen, PA5-80191), Plcβ2 (1:100; Signalway Antibody, 48323). After washing with PBS three times for 5 minutes, the cells were incubated with species specific secondary antibodies (1:200; Abcam, ab150083 and ab150077) coupled with different fluorescent groups at 37°C for 1 hour. DAPI (Solarbio) was used for room temperature staining for 5 minutes to visualize the nucleus. An anti-fluorescence quenching sealing agent was used to cover the sample, and the sample was observed and photographed using a fluorescence microscope (Olympus, IX73).

### RNA silence and heterologous expression

MGFs were transfected with 50 nM Tas2r143-siRNA or non-target-siRNA (siCtrl) (synthesized by Hippo Biotechnology, Primer sequence: mTas2r143 siRNA sense CC UGGCUUGCCAUCUUCUACUTT; mTas2r143 siRNA antisense AGUAGAAGAU GGCAAGCCAGGTT) using lipofectamine 2000 (0.5 μl/well, Thermo Fisher Scientific Inc., 11668019) according to the manufacturer’s instructions. The silencing efficiency of Tas2r143 was confirmed using qRT-PCR.

To determine whether Tas2r143 can be activated by salicin, a construct with heterologous expression of Tas2r143 and chimeric G protein Gα16gust44 was constructed in HEK293 cell line to couple taste receptor activation to Ca^2+^ mobilization. The coding regions of Tas2r143 were amplified from cDNA from WT mouse taste bud tissue (kindly provided by Peihua Jiang, Monell Chemical Senses Center), and the resulting product was subcloned into expression plasmid pcDNA™3.1/Zeo(+) (Thermo Fisher Scientific Inc., V86020). After HEK293 in 96-well plates were cultured to a cell fusion degree of 90%, the Tas2r143 (100 ng/well) construct and the chimeric G protein Gα16gust44 construct (100 ng/well; provided by Peihua Jiang) were instantly transfected into cells using lipofectamine 2000 (0.5 μl/well, Thermo Fisher Scientific Inc., 11668019) as described earlier (13). The calcium flux was measured 24 hours after transfection.

### Calcium imaging

A typical bitter taste signal can lead to an increase in intracellular calcium (Ca^2+^). This Ca^2+^ reaction is usually elicited via gustducin/PLCβ2 cascades, activation of voltage gated channel and transmitter release (13, 17). Therefore, we investigated the activation of taste signaling by monitoring calcium mobilization. MGFs (1 per well × 10^4^ cells) were cultured overnight in 96-well black plates (Corning) with transparent bottoms. Cells were washed twice with Dulbecco’s Phosphate Buffered Saline (DPBS; Hyclone) containing calcium and magnesium, and then loaded with Fluo-4 AM (Thermo Fisher Scientific Inc., F14201) and F-127 (Thermo Fisher Scientific Inc., P3000MP) in the dark at room temperature for 1 hour. After washing with DPBS three times, the cells were incubated in darkness for another 30 minutes to completely de-esterify the dye. For single-cell calcium imaging, cells were examined using standard GFP filters (Olympus, IX83), and images were captured every 1 second for 140 seconds (inject salicin into the well at~10 seconds). 10 µM ATP (Solarbio) and DPBS were used as positive and negative controls, respectively. The baseline normalization curve of fluorescence intensity (F/F0; F0 was determined as the average of the first 5 readings) was drawn to time using GraphPad 8.0 (GraphPad Software Inc.).

### Cell viability

MGFs were treated with a gradient concentrations of salicin (1-20 mM) and LPS (10-200ng/mL) for 12 hours. Cell Count Kit 8 (CCK-8; ApexBio) solution (DMEM: CCK-8=9:1) was added to culture at 37°C for 1 hour. The optical densities (OD) at 450 nm were measured using an enzyme-linked immunosorbent assay to reflect cell viability.

### LPS induction

MGFs were cultured until near fusion, and LPS (200 ng/mL; Sigma, L2880) and/or D (-)salicin (10 mM; Sigma, S0625) were then added for 12 hours. Cells were collected and the lysates were used for further experiments.

### RT-PCR and qRT-PCR

According to the manufacturer’s instructions, total RNA was extracted from fresh mouse gingival tissue and cultured MGFs using TRIzol reagents (Life Technologies) and MiniBest Universal RNA Extraction Kit (TaKaRa, 9767), respectively. After air drying, the sediment was resuspended in water without RNase. The purity and concentrations of isolated RNA were determined with a nanodrop 2000 spectrophotometer (Thermo Fisher Scientific Inc.). RT reagent Kit with gDNA Eraser (TaKaRa, RR047A) was used to generate cDNA. Aliquot without reverse transcriptase was prepared as a negative control. PrimeSTAR ® Max DNA Polymerase (TaKaRa, 045A) was used for RT-PCR to detect the presence of Tas2r143 and its downstream molecules. TB Green Premium Ex Taq II kit (TaKaRa, RR820A) was used to detect the mRNA level of targeted genes. The transcription levels of inflammatory factors (Il17, Il1β, and Tnfα) and chemokine (Cxcl1, Cxcl2 and Cxcl5) were determined. All primers were designed by Primer-BLAST online (https://www.ncbi.nlm.nih.gov/tools/primer-blast/) and are shown in **Appendix Table 1**. The expression levels of target genes after normalization to glyceraldehyde-3- phosphate dehydrogenase (*Gapdh*) in MGFs were calculated using the 2^- △△CT^ method.

### Measurement of alveolar bone loss

Maxillae were dissected from WT and Gnat3^−/−^ mice. The maxillae samples were soaked in a 10% sodium hypochlorite solution for 30 minutes. The soft tissues were removed, and the maxillae were stained in a 2% methylene blue solution for 1 minute. After repeated cleaning with deionized water, images were collected under a stereomicroscope (Olympus, SZX10). Additionally, fixed maxillae were randomly selected and scanned by microCT (μCT 50, Scanco). The X-ray beam was set at 70 kVp and 200 μA. All samples were scanned in the sagittal position at a voxel resolution of 8 μm. The alveolar bone loss (mm) was measured from the cemento-enamel junction (CEJ) to the alveolar bone crest (ABC) of the distal area of M1 and the mesial area of M2 by Image Pro Plus 6.0 (Media Cybernetics, Silver Spring, MD). After 3D reconstruction, the region of interest (ROI) was defined as a rectangular region from the CEJ to the alveolar bone base between M1 and M2. Histomorphometry of trabecular bone at the ROI was assessed by CT-Analyser 1.13 software (Bruker), including bone volume per tissue volume (BV/TV) and trabecular number (Tb.N).

### Histological analyses

Samples were prepared from maxillae for histological analyses. After fixing the samples with 4% paraformaldehyde for 24 hours, decalcification solution (Yobibio, YB2003) was used for demineralization at 4°C, and the solution was changed every two days. Paraffin embedding was carried out one month later. Paraffin-embedded blocks were sectioned at 5μm intervals using a microtome (HM325, Thermo Fisher Scientific, USA). Ly6G^+^ cells in the mouse maxillae were examined by immunohistochemistry (IHC) with a the specific primary antibody (1:500; Abcam, ab238132) and a diaminobezidin (DAB) system (Absin, abs957) according to the manufacture’s instruction. Five high-power fields (hpf) (×400) per each sample at ROI were randomly selected and the positively-stained cells were enumerated by Image Pro Plus 6.0 (Media Cybernetics, Silver Spring, MD).

### Statistical analysis

All data were statistically analyzed by SPSS 26.0 statistical software (SPSS, Inc.). The measurement data were expressed as mean ± standard deviations (mean ± SD). Multiple group comparisons were performed by one-way analysis of variance test followed by Tukey’s test to compare differences between specific groups. Data were considered significantly different if the two-tailed *p* value was <0.05. Sample sizes for each experiment were given in the corresponding figure legends.

## Results

### Mouse gingival fibroblasts express Tas2r143 and taste signaling elements

**Figure 1:**
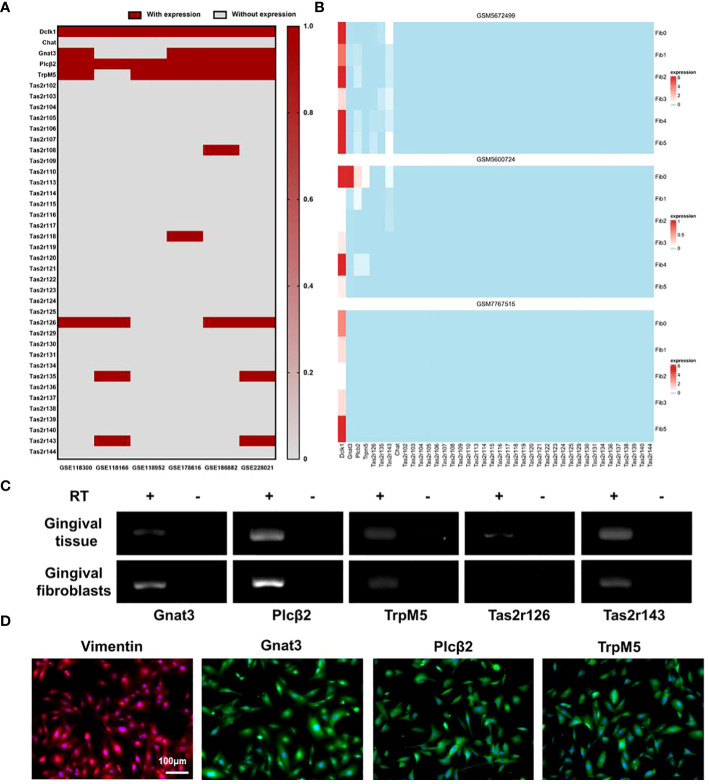
Expressions of SCC markers and taste transduction elements in MGFs. **(A)** Re-analysis of the expression of SCC markers and taste transduction elements in mouse gingivae based on data released by GEO RNA-seq database; **(B)** Re-analysis of the expression of SCC markers and taste transduction elements in MGFs based on data released by GEO scRNA-seq database, Fib 0-5 represents different cell subtypes in MGFs; **(C)** Expression of taste transduction elements in mouse gingivae/MGFs by RT-PCR. RT +/−: with/without reverse transcription; **(D)** Immunofluorescent staining of Vimentin (red), Gnat3 (green), Plcβ2 (green), or TrpM5 (green) in MGFs, respectively. Nuclei were stained by DAPI (blue).

Re-analysis of GEO RNA sequence database identified 6 RNA-seq mouse datasets reporting the expression of taste receptors Tas2r108, -118, -126, -135, -143 and taste pathway elements Gnat3, Plcβ2, TrpM5, as well as SCC markers Dclk1 in the mouse gingival tissue (**Figure 1A**). We further analyzed 3 recent single cell RNA-seq mouse datasets. MGF populations in gingival tissue are shown in **Appendix Figure 1**. In GSM5672499, the expression of Tas2r-126, -135, -143, Gnat3, Plcβ2, and TrpM5 was detected in MGFs; In GSM5600724, the expression of Tas2r143, Gnat3, Plcβ2, and TrpM5 was detected. More importantly, Dclk1 was highly expressed in all 3 studies in MGFs (**Figure 1B**). Because our previous study identified TAS2R16 in human gingival fibroblasts as the specific receptor of salicin, here we further investigated the expression of Tas2r126 and Tas2r143 (human TAS2R16 analogues) and taste signaling elements Gnat3, Plcβ2 and TrpM5 in MGFs. The RT-PCR data showed the expression of Gnat3, Plcβ2, TrpM5, Tas2r126 and Tas2r143 in the controlled mouse gingival tissue (**Figure 1C**). Consistently, the expression of Gnat3, Plcβ2, TrpM5 and Tas2r143 was observed in the primary MGFs, while the expression of Tas2r126 was not detected (**Figure 1C**). Immunofluorescent staining further confirmed the expression of taste signaling elements Gnat3, Plcβ2 and TrpM5 in MGFs (**Figure 1D**). Similarly, the immunofluorescence staining of mouse taste buds serves as a positive control for these elements (**Appendix Figure 2**).

### Salicin induces taste signaling via Tas2r143 in mouse gingival fibroblasts

**Figure 2:**
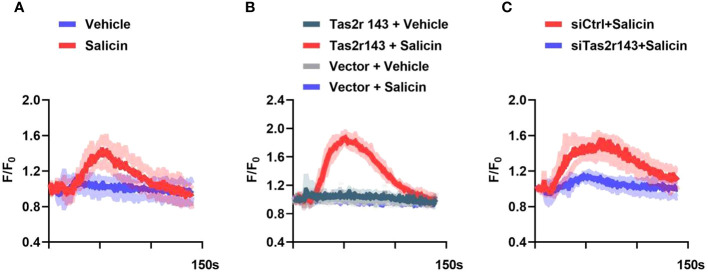
Salicin induces intracellular calcium response in MGFs via Tas2r143. **(A)** Intracellular calcium response of MGFs to 10 mM salicin; **(B)** Calcium responses of HEK293 cells transfected with Tas2r143 and chimeric reporter G protein (Gα16Gust44) to salicin (10 mM); **(C)** Effects of Tas2r143 silencing on the calcium response of MGFs to salicin (10 mM). Data are presented as mean (red/blue/green/gray line) ± standard deviation of 3 independent experiments (shadow). Vector: blank expression plasmid control; siCTRL: non-target control for siRNA silencing; siTas2r143: Tas2r143 knockdown by siRNA silencing.

We investigated whether salicin was able to elicit calcium response via Tas2r143. We selected 10mM concentration of salicin with the most obvious calcium influx reaction for subsequent experiment by setting concentration gradients of 1,5,10 mM salicin (**Appendix Figure 3**). Single cell calcium imaging showed that salicin induced a strong increase of intracellular Ca^2+^ in MGFs (**Figure 2A**). Consistently, an increase of intracellular Ca^2+^ was also detected in HEK293 cells with heterologous expression of Tas2r143 upon salicin stimulation (**Figure 2B**). We further knocked down Tas2r143 in MGFs using siRNA, and the high efficiency of Tas2r143 gene silencing was observed through fluorescence microscopy and qRT-PCR (**Appendix Figure 4**). We found that the calcium flow response of MGFs to salicin was significantly weakened (**Figure 2C**), indicating that salicin elicited taste-like response in MGFs via Tas2r143.

### Salicin inhibits LPS-induced chemokine expression in MGFs dependent on Tas2r143

**Figure 3:**
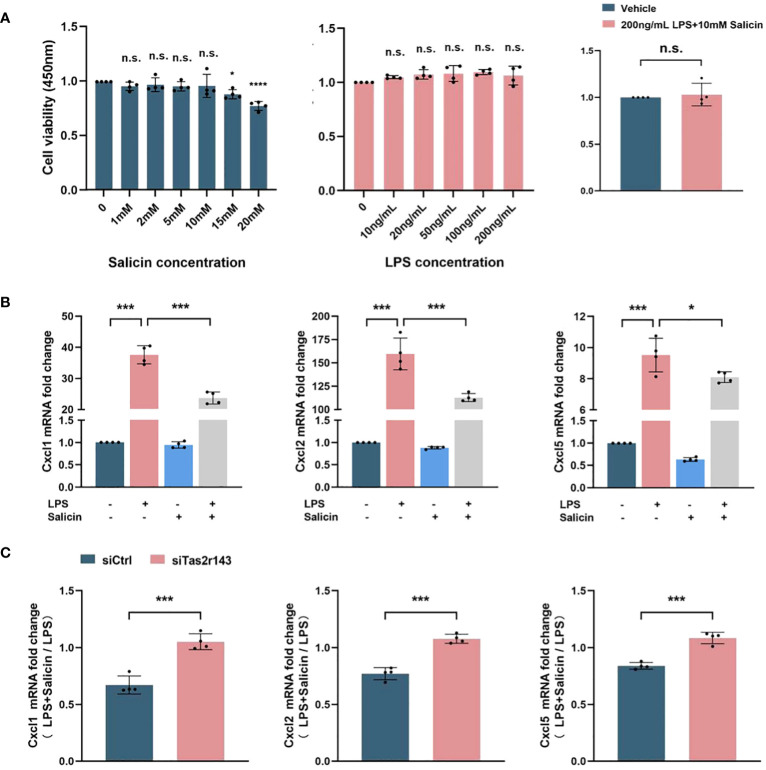
Salicin inhibits LPS-induced chemokine expression by MGFs via Tas2r143. **(A)** The effect of salicin/LPS/salicin+LPS on the cell viability of MGFs; **(B)** Effects of salicin (10mM) on the expression of chemokines in MGFs induced with LPS or vehicle, the truncated Y-axis represents data with significant numerical differences that have been omitted; **(C)** Effects of Tas2r143 silencing on the expression of chemokines in MGFs treated with either LPS (200 ng/mL) or LPS (200 n g/mL)/salicin (10mM). The transcriptional levels were calculated by normalizing the expression level of LPS/salicin group to MGFs treated with LPS alone. Data are presented as mean±SD. *P < 0.05, ***P < 0.001; n.s., not significant compared with control.

We investigated whether the salicin-induced calcium response had impact on chemokine expression of MGFs. 10 mM salicin alone, 200ng/mL LPS alone and the combination of them showed no significant cytotoxicity on MGFs after 12 hours treatment (**Figure 3A**). Therefore, these concentrations were used for the subsequent experiments. MGFs were induced by LPS, and addition of salicin significantly down-regulated the expression of neutrophil chemokines CXCL1, CXCL2 and CXCL5 in vitro (**Figure 3B**). However, the inhibitory effects of salicin on the expression of CXCL1, CXCL2 and CXCL5 in LPS-induced MGFs were abolished after Tas2r143 gene silencing (**Figure 3C**). To eliminate the off target effect of siRNA, we repeated the above experiment using siRNA synthesized from different sequences and obtained similar results (**Appendix Figure 5**).

### Salicin inhibits periodontitis via taste transduction pathway

**Figure 4:**
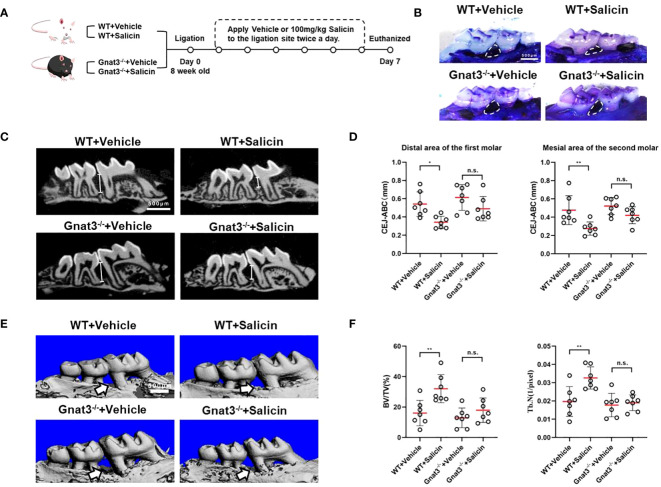
Salicin inhibits alveolar bone resorption in periodontitis mice via taste transduction pathway. **(A)** Schematic diagram of mouse model and treatment; **(B)** Methylene blue staining of maxillae, the white arrows indicate the area of alveolar bone resorption; **(C)** Micro-CT analysis, the white line segment represents the CEJ-ABC; **(D)** Quantitative analysis of CEJ-ABC; **(E)** Representative images of micro-CT reconstruction, the white arrow indicates the area of alveolar bone resorption; **(F)** Quantitative analyses of trabecular microarchitecture. N=7 mice per group. Data are presented as mean±SD. *P < 0.05, **P < 0.01; n.s., not significant; CEJ, cementoenamel junction; ABC, alveolar bone crest.

We constructed a mouse model of periodontitis limited to M1 and M2 successfully (**Appendix Figure 6**). We further investigated whether salicin inhibits periodontal inflammation via taste-like signaling (**Figure 4A**). Topical application of salicin significantly inhibited periodontal bone loss as reflected by reduced alveolar bone loss between the 1^st^ and 2^nd^ maxillary molars (**Figure 4B**), decreased CEJ-ABC distance (**Figures 4C, D**), and improved microarchitecture of the alveolar bone (**Figures 4E, F**) compared with the vehicle-treated periodontitis mice. However, the protective effects of salicin on periodontitis were not observed in the Gnat3^-/-^ mice (**Figures 4B–D**).

**Figure 5:**
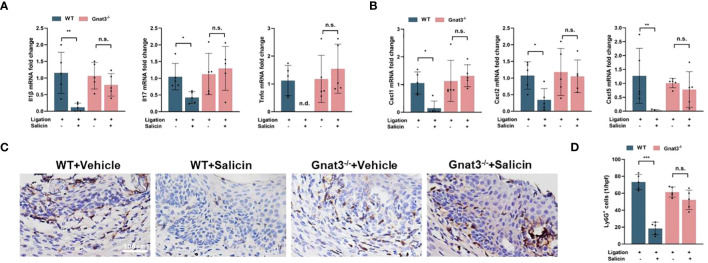
Salicin inhibits the expression of pro-inflammatory cytokines and chemokines in mouse periodontitis through taste transduction pathway. **(A, B)** Expression of inflammatory cytokines and chemokines in mouse gingival tissue; **(C, D)** Representative images of neutrophil (Ly6G^+^) infiltration in gingival tissue and quantitative analysis. N=5 mice per group. Data are presented as mean±SD. *P < 0.05, **P < 0.01, ***P < 0.001; n.s., not significant; n.d., not detected.

We further investigated whether Gnat3-knockout had impact on the anti-inflammatory effect of salicin on periodontitis. Data obtained from qRT-PCR analyses of gingival tissue showed that salicin treatment significantly inhibited the expression of proinflammatory factors in the WT-periodontitis mice, including Il-1β, Tnf-α, Il-17. However, salicin treatment had no significant effects on the expression of these proinflammatory factors in the Gnat3^-/-^ periodontitis mice (**Figure 5A**). We further quantified the expression of neutrophil chemokines including Cxcl1, Cxcl2 and Cxcl5. Salicin treatment significantly inhibited the expression of CXCL1, CXCL2 and CXCL5 in the gingival tissue of WT-periodontitis mice, while these inhibitory effects of salicin were abolished in the Gnat3^-/-^ periodontitis mice (**Figure 5B**). Consistently, neutrophil infiltration in the mouse gingivae was significantly reduced in the salicin-treated WT-periodontitis mice, while this inhibitory effect was not observed in the Gnat3^-/-^ periodontitis mice (**Figures 5C, D**).

## Discussion

G-protein coupled receptors (GPCRs) are widely expressed in a variety of cells and tissues, and are involved in multiple physiological and pathological processes of mammals (18, 19). TAS2Rs are a group of unique GPCR, and when expressed in the non-gustatory tissues, they usually induce suppressing effects via taste transduction pathway to counter overreaction of host such as sustained inflammation and bone resorption (20, 21). These extra-gustatory taste-like cells are commonly defined as SCCs or tuft cells (22). The development and progression of periodontitis involve unresolved inflammation and alveolar bone resorption (2). Our recent study has shown that bitter agonist salicin can activate TAS2R16 in human gingival fibroblasts in vitro, and thus promotes inflammation resolution by antagonizing NF-κB signaling cascade via taste transduction pathway (14). Mouse Tas2r 143 and 126 are structurally and functionally similar to human TAS2R16 (23). The current study demonstrated the expression of Tas2r143 and taste transduction elements Gnat3, Plcβ2 and TrpM5 in MGFs. However, Tas2r126 was only detected in gingival tissue but not in gingival fibroblasts, suggesting its possible presence in other cell types. More importantly, bitter agonist salicin was able to activate the taste transduction pathway via Tas2r143, and thus inhibited chemokine expression and consequently alleviated periodontitis.

The expression of taste receptors and taste pathway elements has been indicated in gingival tissues of human and mice (13, 14). A recent single-cell RNA-seq study has identified a fibroblast subset in human gingiva with high expression of SCCs markers Plcβ2 and DCLK1, as well as bitter taste receptors TAS2R4 and TAS2R14, making this subset a potential type of SCCs (3). However, whether MGFs also possess SCC-like potential and participate in the regulation of periodontal homeostasis via taste transduction pathway has yet to be investigated. We first re-analyzed sequence data deposited in the GEO RNA-seq database (24, 25). We found that the expression of Tas2r143 was only reported in 2 of the 6 RNA-seq databases, and only one of these databases reported the expression of Gnat3. In addition, simultaneous expression of Tas2r143 and Gnat3 in mouse gingival fibroblasts was reported in 2 of the 3 sets of single-cell RNA-seq database. This discrepancy may be attributed to difference in sampling method, sequencing depth, and relatively low expression level of specific taste-transduction elements in mouse gingivae. Therefore, we further validated the expression of Tas2r143 and Gnat3, Plcβ2 and TrpM5 in MGFs by RT-PCR and immunofluorescence. More importantly, we demonstrated that bitter taste agonist salicin signaled via Tas2r143 to elicit taste-like intracellular calcium flow in MGFs, further indicating the chemosensory potential of MGFs.

Activation of SCCs/tuft cells has been reported to contribute to homeostasis of resided microenvironment (26). Intestinal tuft cells are a typical group of chemosensory cells that express TAS2Rs. Recent studies have shown that tuft cells can sense eukaryotic parasites via TRPM5 and sense microbiota-derived succinate via Sucnr1, thus initiating type II immunity (27, 28). In addition to intestine, SCCs in the respiratory tract can sense acyl–homoserine lactones (AHLs) produced by Gram-negative bacteria via bitter taste signaling, thus inducing local inflammatory response to combat bacterial invasion (29); SCCs in the extrahepatic bile duct can sense bile acid, regulate neutrophil influx, and thus limit inflammation (30); SCCs in the gallbladder can sense microbial metabolites propionate and initiate type 2 immunity (31). Similarly, bitter compounds can exert anti-inflammatory effects via taste transduction pathway. TAS2Rs located in human airway epithelial cells can be activated by various bitter compounds such as denatonium and thujone, leading to an increased ciliary beat frequency and enhanced removal of harmful inhaled materials (32). Bitter compounds chloroquine or quinine can inhibit immune cell chemotaxis by activating TAS2Rs, and thus block allergic airway inflammation (33). Consistently, our study showed that bitter compound salicin was able to agonize Tas2r143 expressed by MGFs, eliciting taste-like signaling with subsequent inhibition of chemokines, and thus contributed to the alleviation of periodontal inflammation and bone destruction.

Gingival fibroblasts as the accessory immune cells in gingivae can secret chemokines such as CXCL1, CXCL2 and CXCL5, attracting neutrophils to periodontium and crevicular fluid to combat microbial evasion (34, 35). However, excessive recruitment of neutrophils amplifies the inflammatory response by producing inflammatory factors such as IL-1β and TNF-α, and releasing enzymes or metabolites such as reactive oxygen species, granular enzymes and MMPs, further aggravating periodontal inflammation and osteoclastic bone resorption (36, 37). Inhibition of excessive migration and sustained infiltration of neutrophils is beneficial for the resolution of periodontitis (38, 39). For example, aminoguanidine, a nitric oxide synthase inhibitor, significantly reduces neutrophil infiltration in periodontitis mice and inhibits periodontal destruction (40). The decrease in neutrophil recruitment and activation can alleviate inflammation-induced damage, but at the cost of reducing bacterial killing and wider spread of infection (41). The current study demonstrated for the first time that salicin was able to suppress the chemokine expression by MGFs, and this inhibitory activity of salicin depended on the activation of Tas2r143 and its downstream gustducin-mediated taste transduction pathway. More importantly, topical application of salicin significantly suppressed neutrophil infiltration and alleviated periodontal destruction in WT mice rather than Gnat3^-/-^ mice, further confirming the SCC-like activity of gingival fibroblasts in the development and resolution of periodontitis. It should be noted that our previous study showed aggravated periodontal bone loss in the ligature-induced Gnat3^-/-^ mice as compared to the WT controls (13). However, although slightly increased alveolar bone loss was observed in Gnat3^–/–^ periodontitis mice as compared to WT mice in the current study, no statistical significance was observed. This discrepancy was likely due to the different LIP model used in the current study. Our previous study induced periodontitis by tying silk ligatures around the left maxillary second molar of mice to maximize microbial colonization, as we hypothesized that Gnat3^-/-^ mice harbored distinct microbiota that induced more severe periodontal bone loss (13). However, the current study was focused on the anti-inflammatory effect of salicin via taste receptor and its downstream pathway, we used a simplified LIP method by placing the ligation between the first (M1) and second (M2) maxillary molars, allowing relatively less bacterial accumulation only in the interdental space between M1/M2 (16) and thus reduced the confounders that might be caused by varied microbiota in different genetic background. As a result, we observed that salicin significantly alleviated periodontitis in WT mice with respect to gingival inflammation and alveolar bone loss, and this anti-periodontitis effects were significantly abolished in Gnat3^-/-^ mice.

In summary, the current study has demonstrated the SCC activity of gingival fibroblasts in the context of periodontitis. Activation of Tas2r143/gustducin taste transduction pathway suppresses chemokine expression and the subsequent neutrophil infiltration, and consequently alleviates periodontal destruction. Findings from this study will not only advance our understanding of gingival fibroblasts in the maintenance of periodontal homeostasis, but also provide a promising approach to the resolution of periodontal inflammation via stimulating the “SCC-like” function of gingival fibroblasts. However, as Gnat3^-/-^ mice we used in the current study were global knockout mice, other cell types in gingiva such as epithelial, endothelial and immune cells may also express Gnat3 and Tas2r143, and thus mediate the action of salicin. MGF-specific Gnat3 knockout mouse are still needed to further explore the potential mechanisms in depth, and future clinical study is also needed to validate the effectiveness of salicin in the management of periodontitis.

## Data availability statement

The original contributions presented in the study are included in the article/**Supplementary Material**. Further inquiries can be directed to the corresponding author.

## Ethics statement

The animal study was approved by Ethics Committee of West China Hospital, Sichuan University (WCHSIRB-D-2021-071). The study was conducted in accordance with the local legislation and institutional requirements.

## Author contributions

ZZha: Writing – review & editing, Writing – original draft, Visualization, Validation, Software, Resources, Methodology, Investigation, Formal analysis, Data curation, Conceptualization. ZZho: Writing – review & editing, Visualization, Validation, Supervision, Software, Project administration, Methodology, Investigation, Formal analysis, Data curation, Conceptualization. JL: Writing – review & editing, Investigation, Data curation. LWZ: Writing – review & editing, Validation, Supervision, Resources, Project administration. XP: Writing – review & editing, Validation, Supervision, Resources, Project administration. LZ: Writing – review & editing, Supervision, Project administration. XZ: Writing – review & editing, Validation, Supervision, Resources, Project administration, Methodology. XX: Writing – review & editing, Validation, Supervision, Resources, Project administration, Methodology, Funding acquisition, Conceptualization.
