# Supplementary material for: Salicin alleviates periodontitis via Tas2r143/gustducin signaling in fibroblasts
Source: Front Immunol. 2024 Mar 28;15:1374900. doi: 10.3389/fimmu.2024.1374900 (PMC11007171; doi:10.3389/fimmu.2024.1374900)
Supplement: Supplementary file 1 [file DataSheet_1.docx]

**Supplemental Appendix:**

**Salicin alleviates periodontitis via Tas2r143/gustducin in fibroblasts**

Zhiying Zhang^1,2,#^, Zhiyan Zhou^3#^, Jiaxin Liu^1,2^, Qiang Guo^1^, Liwei Zheng^1,4^, Xian Peng^1^, Lei Zhao^5^, Xin Zheng^1,2^, Xin Xu^1,2,*^

^1^ The State Key Laboratory of Oral Diseases and National Clinical Research Center for Oral Disease, West China Hospital of Stomatology, Sichuan University, Chengdu, Chengdu 610041, P.R. China

^2^ Department of Cariology and Endodontics, West China Hospital of Stomatology, Sichuan University, Chengdu 610041, P.R. China

^3^ Department of Cariology and Endodontics, School and Hospital of Stomatology, Cheeloo College of Medicine, Shandong University & Shandong Key Laboratory of Oral Tissue Regeneration & Shandong Engineering Research Center of Dental Materials and Oral Tissue Regeneration & Shandong Provincial Clinical Research Center for Oral Diseases

^4^ Department of Pediatric Dentistry, West China Hospital of Stomatology, Sichuan University, Chengdu 610041, P.R. China

^5^ Department of Periodontology, West China Hospital of Stomatology, Sichuan University, Chengdu 610041, P.R. China

#Authors contributing equally.

* Corresponding authors: **Xin Xu,** State Key Laboratory of Oral Diseases, National Clinical Research Center for Oral Diseases, West China Hospital of Stomatology, Sichuan University, No.14, Section 3, Renmin Road South, Chengdu, Sichuan, China. Email: xin.xu@scu.edu.cn.

**Appendix Figure Legends**


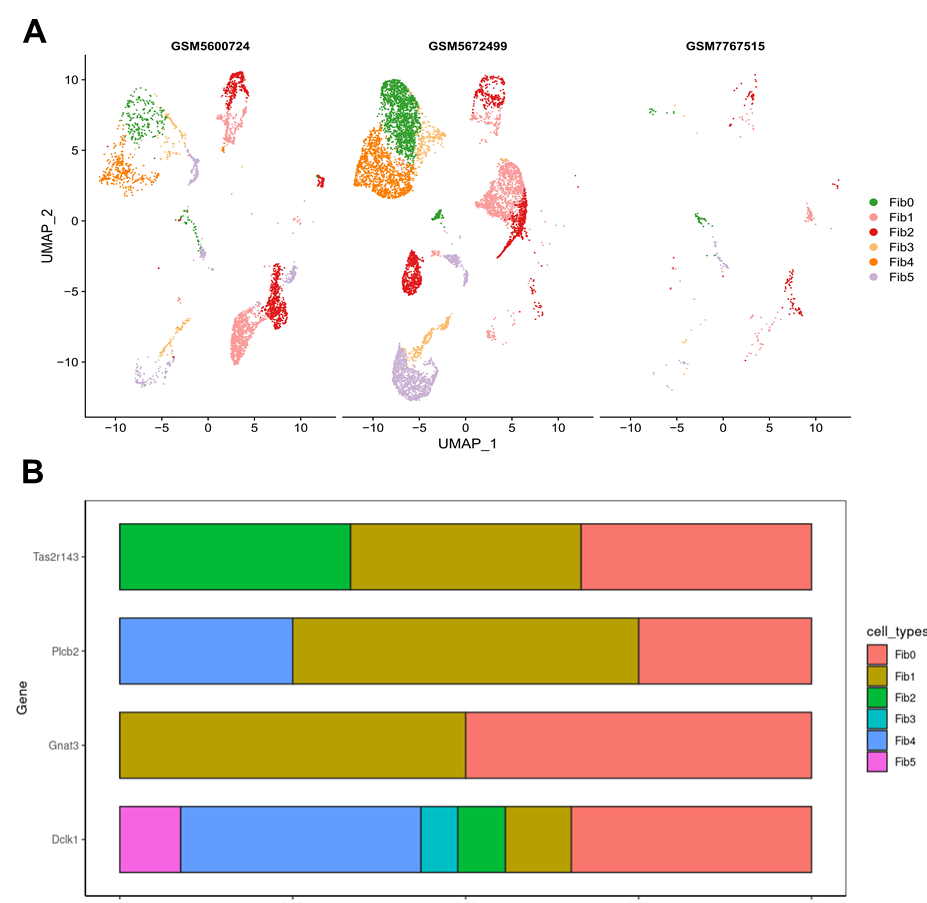


**Appendix Figure 1. MGF populations in gingival tissue.** UMAP (A) and proportion plots (B) of MGF populations in gingiva tissue (n=3). MGFs were annotated using a combination of reference based (SingleR) and manual annotation. Refer to STAR Methods for statistical tests used. Fib 0-5 represents different cell subtypes in MGFs.


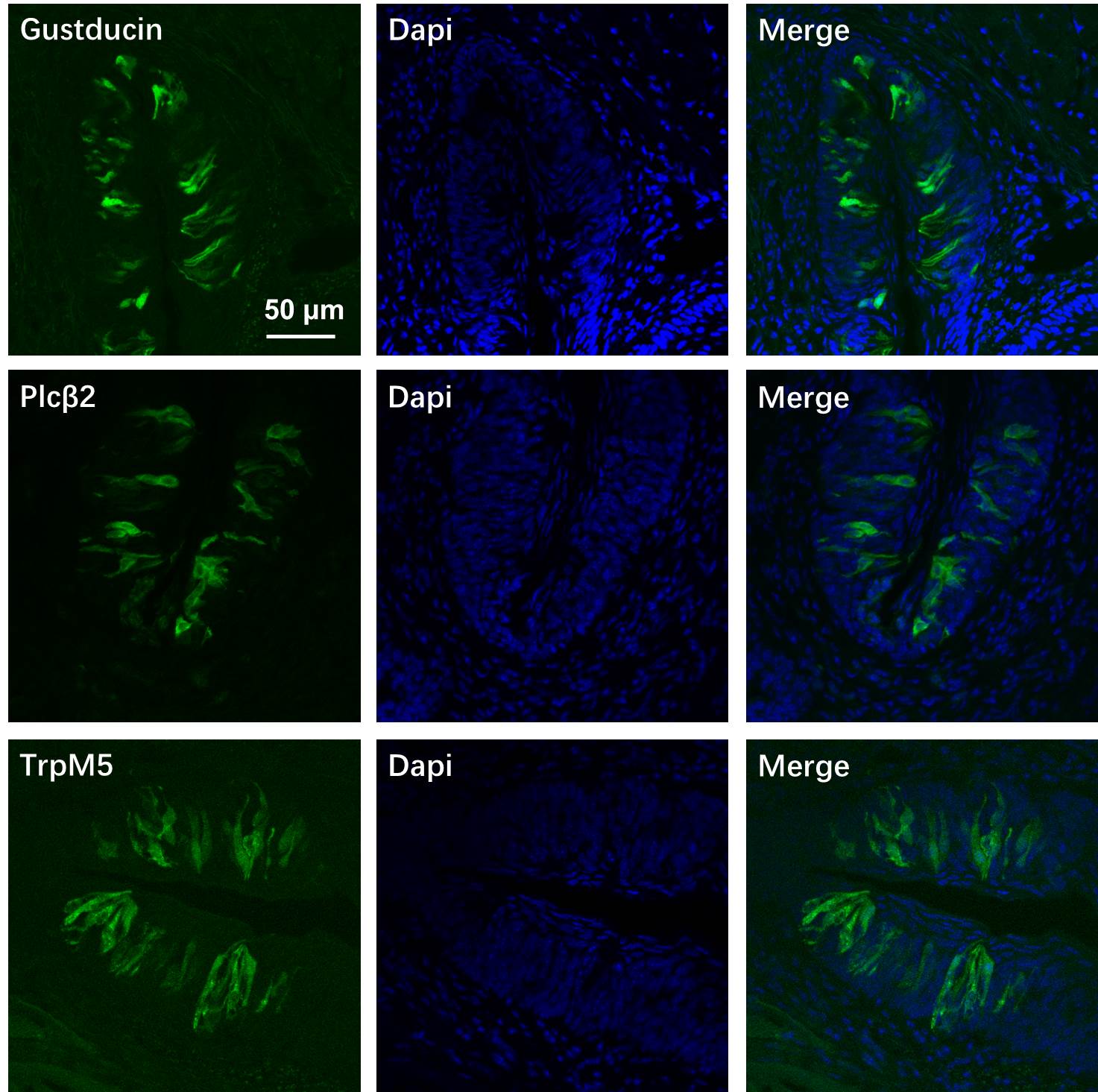


**Appendix Figure 2. Immunofluorescent staining of Gnat3 (green), Plcβ2 (green), or TrpM5 (green) in taste buds, respectively.** Nuclei were stained by DAPI (blue).


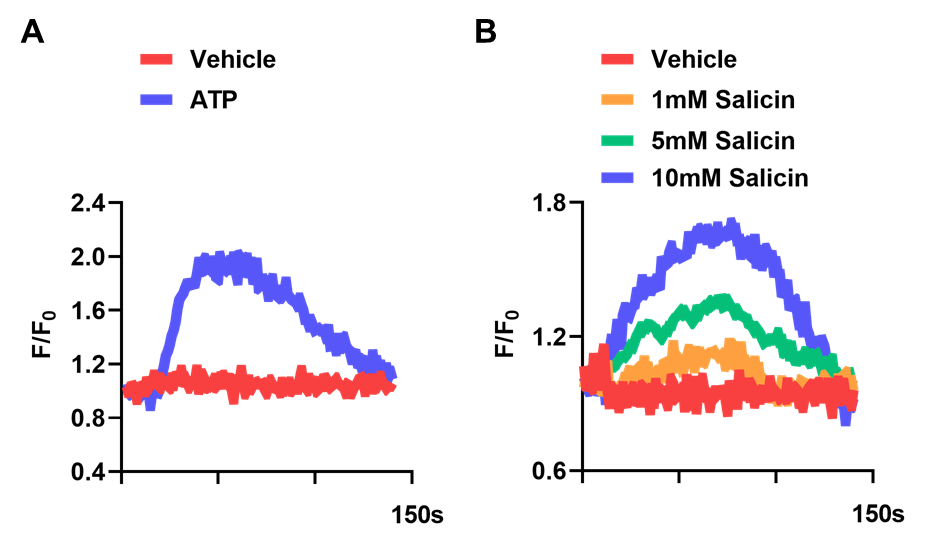


**Appendix Figure 3. Salicin induces intracellular calcium response in MGFs.** (A) Calcium response curves of MGFs to 10 μM ATP as positive control and vehicle as negative control; (B) Intracellular calcium response of MGFs to 1,5,10 mM salicin.

***Tas2r143 transfection***

MGFs were transfected with 50 nM Tas2r143-siRNA or non-target-siRNA (siCtrl) (synthesized by Hippo Biotechnology, Primer sequence: mTas2r143 siRNA-1 sense CCUGGCUUGCCAUCUUCUACUTT; mTas2r143 siRNA-1 antisense AGUAGAA GAUGGCAAGCCAGGTT; mTas2r143 siRNA-2 sense GUCCGUUAUCGUGCUC AUGUUTT; mTas2r143 siRNA-2 antisense AACAUGAGCACGAUAACGGACTT), using lipofectamine 2000 (Thermo Fisher Scientific Inc., 11668019) according to the manufacturer's instructions. The transfection efficiency results are shown in Appendix Figure 4.


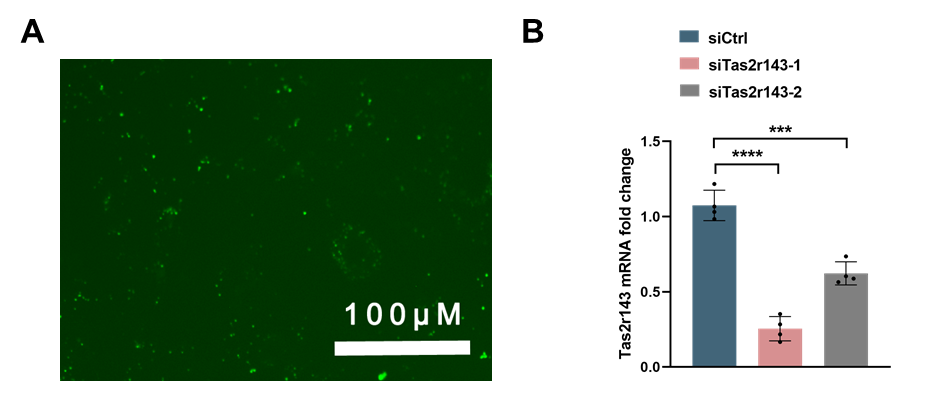


**Appendix Figure 4. Tas2r143 knockout efficiency.** (A) siRNA transfection image under fluorescence microscope. Green fluorescent dots are siRNA; (B) qRT-PCR validation of Tas2r143 knockout efficiency. ****P* < 0.001; *****P* < 0.0001.


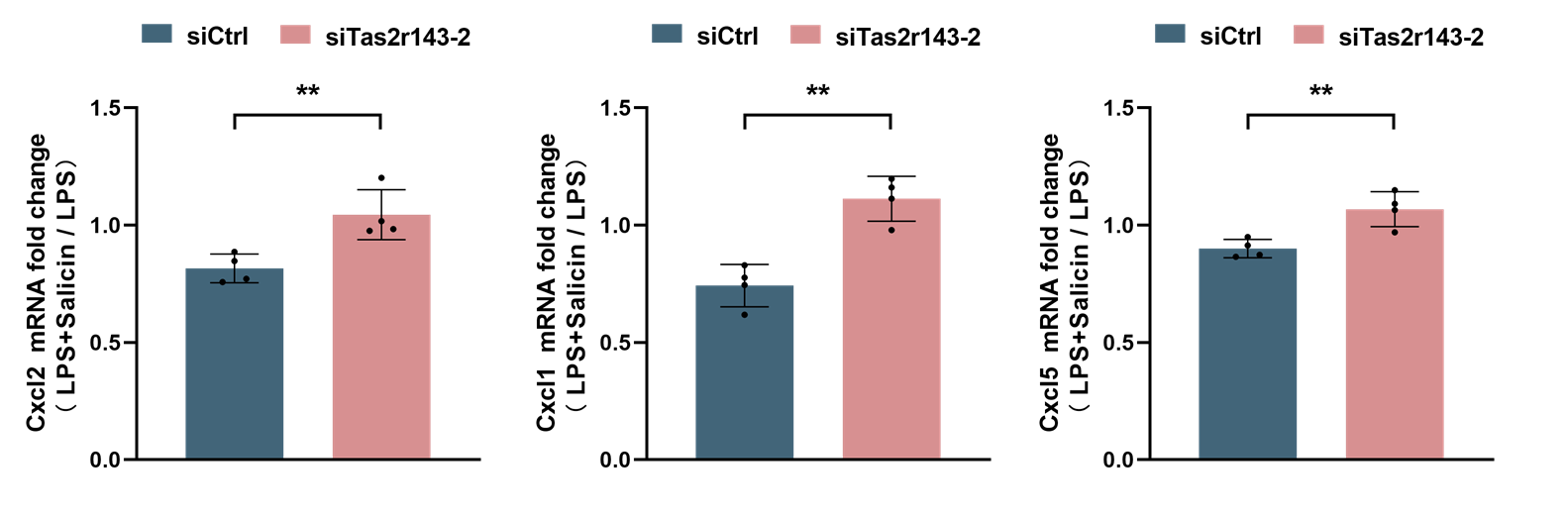


**Appendix Figure 5.** **Salicin inhibits LPS-induced chemokine expression by MGFs via Tas2r143.** Effects of Tas2r143 silencing on the expression of chemokines in MGFs treated with either LPS (0.2 µ g/mL) or LPS (0.2 µ g/mL)/salicin (10mM). The transcriptional levels were calculated by normalizing the expression level of LPS/salicin group to MGFs treated with LPS alone. Data are presented as mean±SD. ***P* < 0.01.


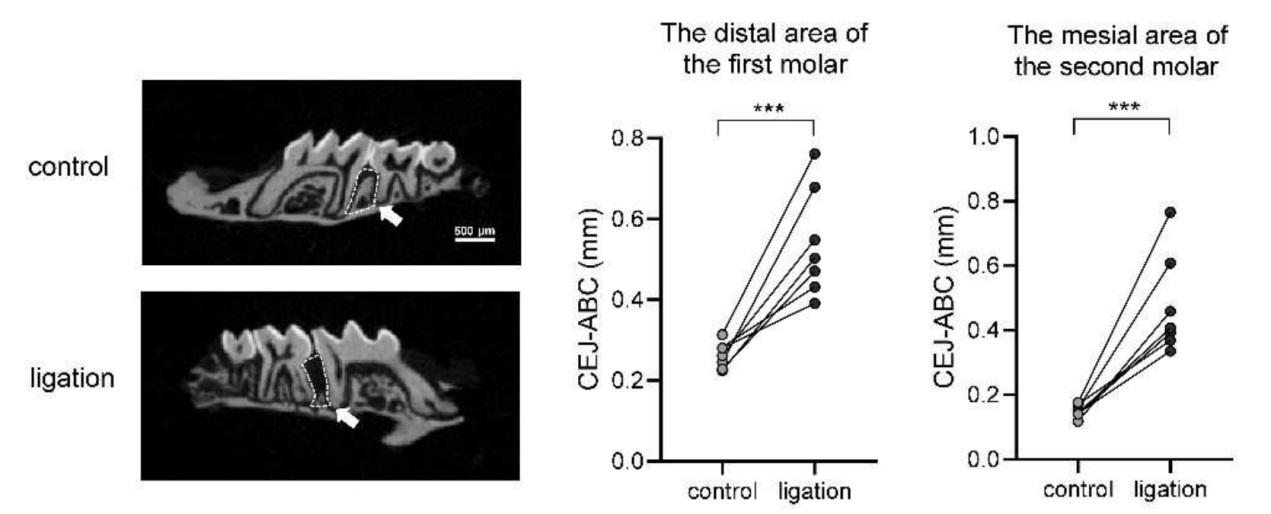


**A**

**B**

**Appendix Figure 6.** **Micro-CT analysis of alveolar bone resorption between maxillary M1-M2 on the ligated and non-ligated sides.** The white arrows indicate the area of alveolar bone resorption between M1-M2. Control, non-ligated side; Ligation, ligated side. ****P*<0.001.

**Appendix Table 1. Primers used in this study**

| Primers | Forward primer (5’ to 3’） | Reverse primer (5’ to 3’) | Species |
| --- | --- | --- | --- |
| *Gnat3* | GAGAGCAAGGAATCAGCCAG | GTGCTTTTCCCAGATTCACC | Mouse |
| *Plcβ2* | CCTGGAGGTGACAGCTTATGA | GCTCCGTGAAGGAAGAGACA | Mouse |
| *TrpM5* | AGCACAATGGAAGGTGTCCTC | GGAAATTGCCAAGCCAGTGAG | Mouse |
| *Tas2r126* | TCCTCTTCAGTTTGGGCACC | CGGACACCAAGATAGAGCCC | Mouse |
| *Tas2r143* | AGAGTGGATGAGGAACCGGA | GCCATGGTATGTGCCTGAGT | Mouse |
| *Cxcl1* | ACTGCACCCAAACCGAAGTC | TGGGGACACCTTTTAGCATCTT | Mouse |
| *Cxcl2* | GAAGTCATAGCCACTCTCAAGG | CCTCCTTTCCAGGTCAGTTAGC | Mouse |
| *Cxcl5* | TGCGTTGTGTTTGCTTAACCG | CTTCCACCGTAGGGCACTG | Mouse |
| *Il1β* | TGCCACCTTTTGACAGTGATG | AAGGTCCACGGGAAAGACAC | Mouse |
| *Il17* | TCAGCGTGTCCAAACACTGAG | CGCCAAGGGAGTTAAAGACTT | Mouse |
| *Tnfα* | CAGGCGGTGCCTATGTCTC | CGATCACCCCGAAGTTCAGTAG | Mouse |
| *Gapdh* | AGGTCGGTGTGAACGGATTTG | GGGGTCGTTGATGGCAACA | Mouse |
