# Supplementary figures and images for: Salicin alleviates periodontitis via Tas2r143/gustducin signaling in fibroblasts
Source: Front Immunol. 2024 Mar 28;15:1374900. doi: 10.3389/fimmu.2024.1374900 (PMC11007171; doi:10.3389/fimmu.2024.1374900)

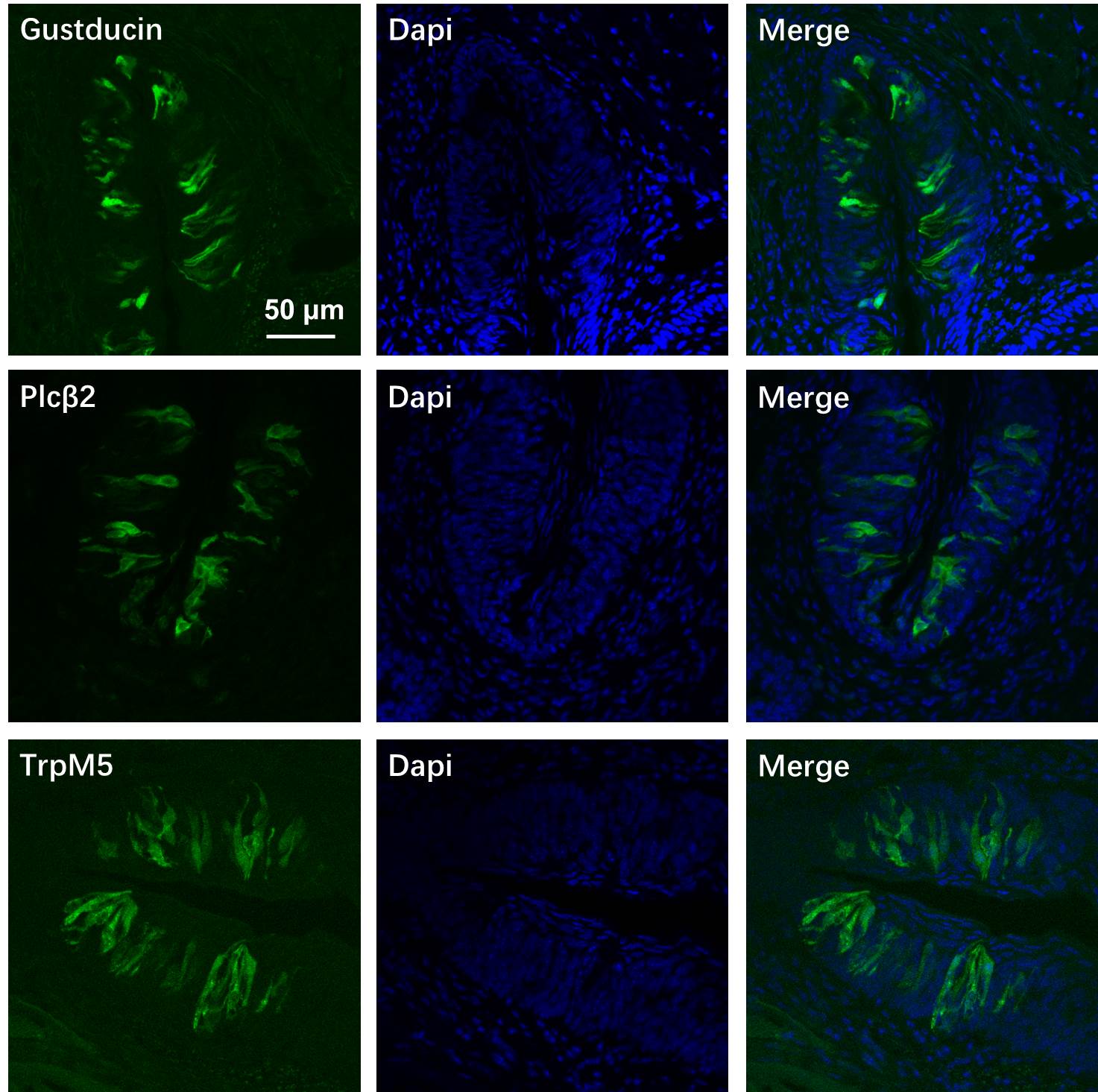

Supplement: Supplementary file 2 [file DataSheet_2.zip › Data Sheet/Appendix/Appendix Figure2/Appendix Figure2.jpg]

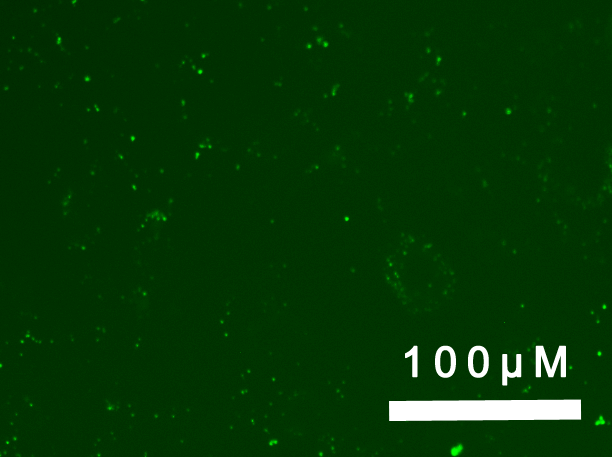

Supplement: Supplementary file 2 [file DataSheet_2.zip › Data Sheet/Appendix/Appendix Figure3/Appendix Figure 3A.tif]

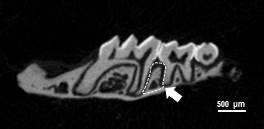

Supplement: Supplementary file 2 [file DataSheet_2.zip › Data Sheet/Appendix/Appendix Figure6/Appendix Figure 5A-C.jpg]

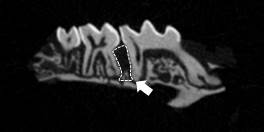

Supplement: Supplementary file 2 [file DataSheet_2.zip › Data Sheet/Appendix/Appendix Figure6/Appendix Figure 5A-L.jpg]

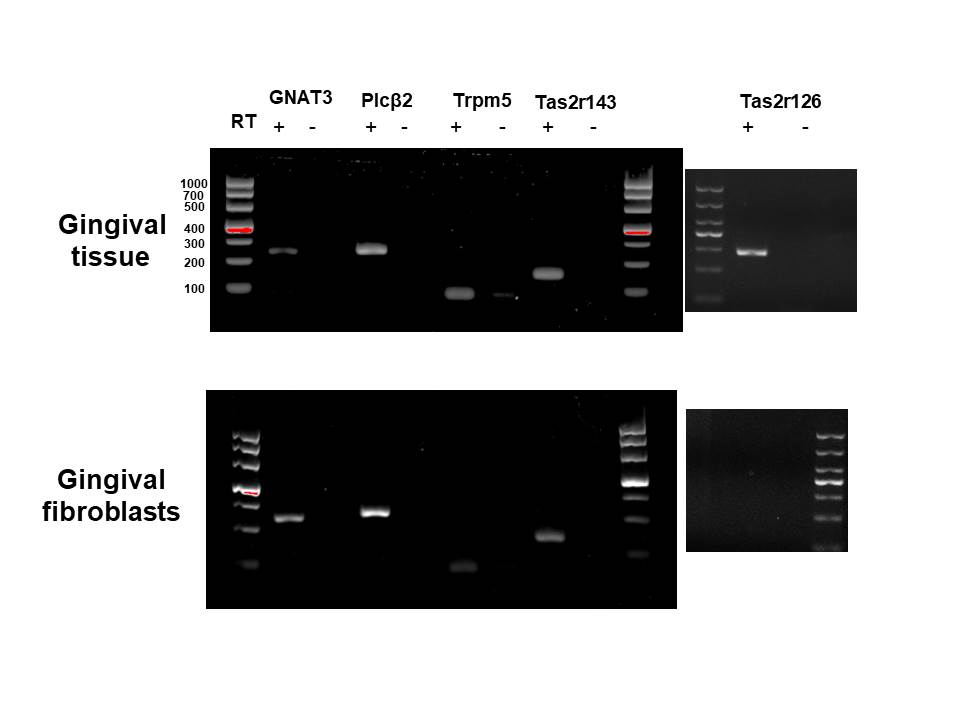

Supplement: Supplementary file 2 [file DataSheet_2.zip › Data Sheet/Figure1/Figure1C.jpg]

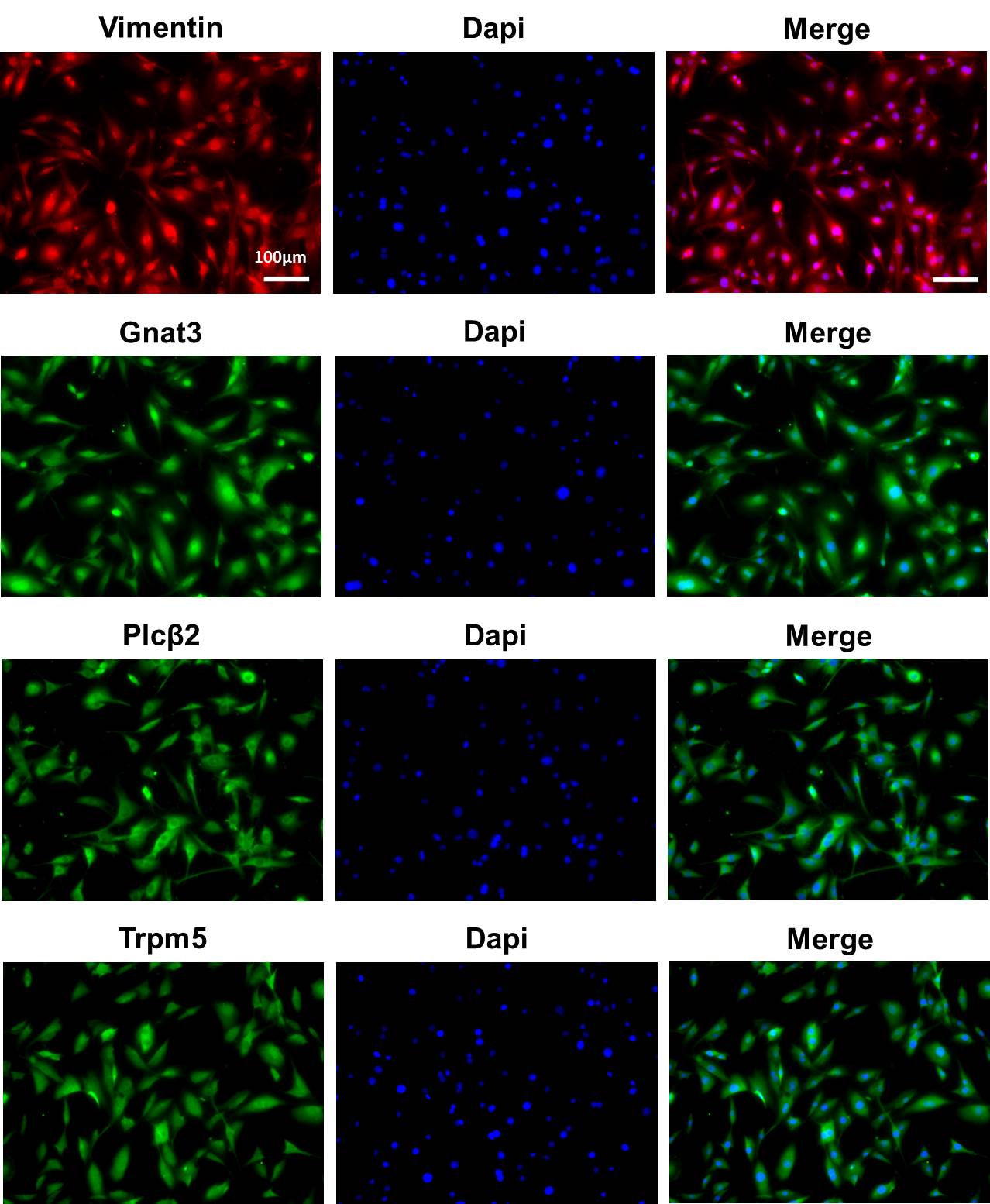

Supplement: Supplementary file 2 [file DataSheet_2.zip › Data Sheet/Figure1/Figure1D.jpg]

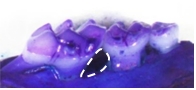

Supplement: Supplementary file 2 [file DataSheet_2.zip › Data Sheet/Figure4/Figure4B/Figure4B-GS.jpg]

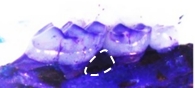

Supplement: Supplementary file 2 [file DataSheet_2.zip › Data Sheet/Figure4/Figure4B/Figure4B-GV.jpg]

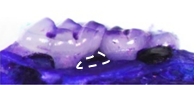

Supplement: Supplementary file 2 [file DataSheet_2.zip › Data Sheet/Figure4/Figure4B/Figure4B-WS.jpg]

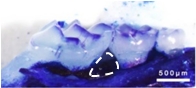

Supplement: Supplementary file 2 [file DataSheet_2.zip › Data Sheet/Figure4/Figure4B/Figure4B-WV.jpg]

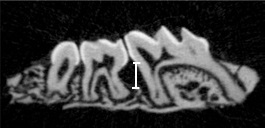

Supplement: Supplementary file 2 [file DataSheet_2.zip › Data Sheet/Figure4/Figure4C/Figure4C-GS.jpg]

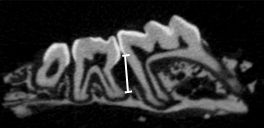

Supplement: Supplementary file 2 [file DataSheet_2.zip › Data Sheet/Figure4/Figure4C/Figure4C-GV.jpg]

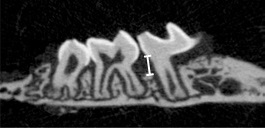

Supplement: Supplementary file 2 [file DataSheet_2.zip › Data Sheet/Figure4/Figure4C/Figure4C-WS.jpg]

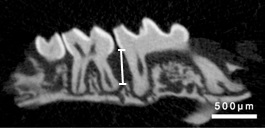

Supplement: Supplementary file 2 [file DataSheet_2.zip › Data Sheet/Figure4/Figure4C/Figure4C-WV.jpg]

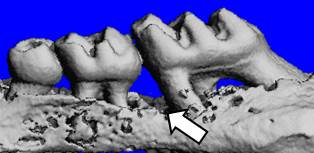

Supplement: Supplementary file 2 [file DataSheet_2.zip › Data Sheet/Figure4/Figure4E/Figure4E-GS.jpg]

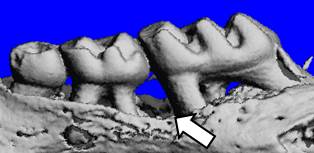

Supplement: Supplementary file 2 [file DataSheet_2.zip › Data Sheet/Figure4/Figure4E/Figure4E-GV.jpg]

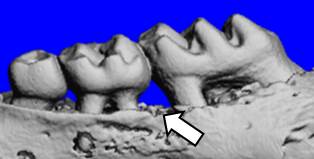

Supplement: Supplementary file 2 [file DataSheet_2.zip › Data Sheet/Figure4/Figure4E/Figure4E-WS.jpg]

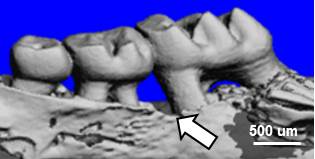

Supplement: Supplementary file 2 [file DataSheet_2.zip › Data Sheet/Figure4/Figure4E/Figure4E-WV.jpg]

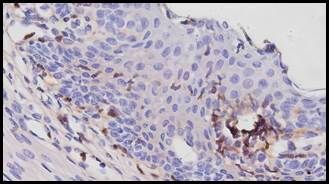

Supplement: Supplementary file 2 [file DataSheet_2.zip › Data Sheet/Figure5/Figure5C/Figure5C-GS.jpg]

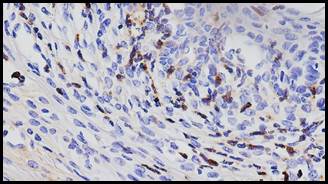

Supplement: Supplementary file 2 [file DataSheet_2.zip › Data Sheet/Figure5/Figure5C/Figure5C-GV.jpg]

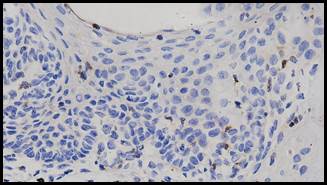

Supplement: Supplementary file 2 [file DataSheet_2.zip › Data Sheet/Figure5/Figure5C/Figure5C-WS.jpg]

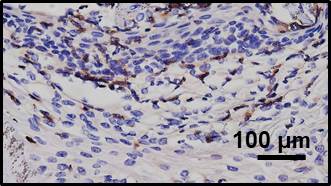

Supplement: Supplementary file 2 [file DataSheet_2.zip › Data Sheet/Figure5/Figure5C/Figure5C-WV.jpg]
